# Transcriptomic Stratification Reveals an FRS2-Associated, Proliferation-Independent Phenotype in MDM2-High Sarcomas

**DOI:** 10.3390/curroncol33070438

**Published:** 2026-07-22

**Authors:** Takao Sakai, Hisaki Aiba, Makoto Yamaguchi, Koji Hagiwara, Hideki Murakami, Hiroaki Kimura

**Affiliations:** Department of Orthopaedic Surgery, Nagoya City University Graduate School of Medical Sciences, Nagoya 467-8601, Japan; teawithtakao@gmail.com (T.S.); marshmallowday1220@gmail.com (M.Y.); matawara.com@gmail.com (K.H.); hmuraka@med.nagoya-cu.ac.jp (H.M.); hikimura@med.nagoya-cu.ac.jp (H.K.)

**Keywords:** *MDM2*, *FRS2*, CINSARC, soft tissue sarcoma, metastasis-free survival, transcriptomic analysis, 12q13-15 amplicon

## Abstract

Sarcomas with *Murine Double Minute 2* (*MDM2*) amplification are considered potential candidates for MDM2-targeted therapy. However, the limited efficacy of MDM2 inhibitor monotherapy suggests that additional biological factors may influence tumor behavior and prognosis. This study investigated the relationship between *MDM2* expression and the Complexity Index in SARComas (CINSARC) transcriptomic signature, a gene expression signature that reflects cell division and chromosomal instability in soft tissue sarcomas. In the public GSE21050 dataset comprising 310 soft tissue sarcomas, the MDM2-low/CINSARC-low subgroup showed the most favorable metastasis-free survival, whereas the MDM2-high/CINSARC-low subgroup had an unfavorable metastasis-free survival comparable to that of the MDM2-high/CINSARC-high group. Among the representative genes in the 12q13-15 region, *fibroblast growth factor receptor substrate 2* (*FRS2*) showed a strong positive correlation with *MDM2* expression, but no significant correlation with CINSARC, suggesting an association independent of proliferative capacity. These findings suggest that proliferation-based risk assessment alone may underestimate the metastatic risk of a subset of MDM2-high sarcomas, and *FRS2* may present a biologically relevant marker of aggressive behavior independent of tumor proliferation.

## Simple Summary

Sarcomas with *murine double minute 2* (*MDM2*) amplification are promising candidates for targeted therapy. However, their treatment response remains limited. To further investigate this limitation, we analyzed a publicly available dataset of 310 soft tissue sarcomas and examined the relationship between *MDM2* expression and the Complexity Index in the SARComas (CINSARC) transcriptomic signature, a gene expression signature that reflects cell division and chromosomal instability. We found that a subset of tumors with high *MDM2* expression had a low proliferative profile (CINSARC-low), but still showed poor metastasis-free survival. Additionally, *fibroblast growth factor receptor substrate 2* (*FRS2*), a gene located near *MDM2*, was strongly coexpressed with *MDM2* and showed no clear association with CINSARC. These findings suggest that proliferation-based risk assessment alone may underestimate the metastatic risk of a subset of MDM2-high sarcomas, and *FRS2* may present a biologically relevant marker associated with aggressive behavior independent of tumor proliferation.

## 1. Introduction

*Murine Double Minute 2* (*MDM2*), located on chromosome 12q15, is an oncogene encoding an E3 ubiquitin ligase, which is the principal negative regulator of the tumor suppressor p53 [1]. MDM2 binds to p53, inhibits its transactivation, promotes nuclear export, and catalyzes p53 ubiquitination, leading to proteasomal degradation [1]. MDM2 inhibitors have been investigated as promising therapeutic options for sarcomas with *MDM2* amplification, including dedifferentiated liposarcomas, intimal sarcomas, and low-grade osteosarcomas [2]. MDM2 inhibitors have been shown to reactivate the p53 pathway, inhibit cell proliferation, and induce apoptosis in both in vitro and in vivo studies [3,4]. However, recent clinical studies on the MDM2 inhibitor, milademetan, have suggested that the efficacy of this monotherapy remains limited. The randomized, multicenter, open-label phase II trial, MANTRA study (RAIN-3201), compared milademetan to trabectedin in adults with unresectable or metastatic dedifferentiated liposarcoma [4]. The study failed to demonstrate statistical superiority in progression-free survival (PFS), with a median PFS of 3.6 months in the milademetan group compared with 2.2 months in the trabectedin group. These findings suggest that the therapeutic response to MDM-2 inhibitors may be limited, and that additional biological factors may influence tumor behavior and prognosis.

The Complexity Index in SARComas (CINSARC) is a 67-gene transcriptomic signature associated with mitotic control and chromosomal integrity that was originally developed as a prognostic tool for metastatic outcomes in soft tissue sarcomas [5,6]. In the original report, CINSARC stratified patients into low- and high-risk groups independent of conventional histological grading and demonstrated prognostic value beyond the FNCLCC grading system [6]. Subsequent studies further established CINSARC as a robust predictor of metastatic risk and survival across multiple sarcoma subtypes and showed that its prognostic value was maintained across different technical platforms [7,8,9,10]. In particular, RNA-sequencing-based validation demonstrated 77% agreement in risk-group classification between microarray and RNA-seq and 88% agreement between frozen and formalin-fixed paraffin-embedded (FFPE) samples [10], whereas NanoString-based assessment on FFPE tissues confirmed strong prognostic performance with a hazard ratio of 4.43 (95% confidence interval, 1.25–15.72) and 84% agreement with matched frozen samples [8]. Recent studies have increasingly reinforced its clinical relevance, including its validation as an independent prognostic factor for disease-free survival in retroperitoneal sarcoma in a combined analysis of single-institution data and the STRASS trial [11] and prospective evaluation in the ongoing phase III CHIC-STS trial for treatment stratification in localized grade 1–2 soft tissue sarcoma [12]. In addition, emerging mechanistic evidence suggests that CINSARC reflects proliferative activity and broader genome-instability-related processes, including association with the alternative lengthening of the telomere pathway in non-translocation-related sarcomas [13].

Collectively, these findings support the clinical and biological relevance of CINSARC as a framework for risk stratification of sarcoma. However, although *MDM2* amplification is associated with tumor growth and proliferative capacity, its specific relationship with CINSARC has not yet been fully investigated. In this study, we investigated the relationship between *MDM2* expression and CINSARC using the publicly available GSE21050 dataset.

## 2. Materials and Methods

### 2.1. Dataset and Platform

A transcriptomic dataset of 310 soft tissue sarcoma cases (GSE21050) was obtained from the Gene Expression Omnibus (GEO) [14]. The dataset included normalized gene expression levels, determination calls indicating the presence or absence of expression, detection *p*-values, and clinical information, including the status of metastasis and follow-up period. The dataset was derived from the Affymetrix Human Genome U133 Plus 2.0 Array (GPL570), which contains more than 54,000 probe sets.

### 2.2. Molecular Stratification by MDM2 and CINSARC

To reduce the arbitrariness in setting thresholds for defining the MDM2-high group, we analyzed transcript abundance data from the Cancer Genome Atlas (TCGA) sarcoma cohort using RNA sequencing by expectation-maximization (RSEM) values [15]. A Gaussian Mixture Model (GMM) was applied to the *MDM2* expression distribution, revealing a distinct bimodal pattern. The GMM mathematically clustered the cohort into a baseline-expression subpopulation (mean RSEM = 1083, representing 71.7% of the cohort) and a high-expression subpopulation (mean RSEM = 26,000, representing 28.3% of the cohort) (Table A1).

Although the GMM-derived boundary identified 28.3% of the cohort as the high-expression group, previous studies have reported the genomic prevalence of *MDM2* amplification in sarcomas to be lower, typically ranging from 13% to 20% [10]. Furthermore, it is well documented that genomic DNA amplification does not always translate to massive mRNA overexpression because of biological uncoupling at the transcriptomic level [16,17]. Therefore, rather than attempting to capture all genomically amplified cases (such as all dedifferentiated liposarcomas), we specifically extracted a subset that exhibited extreme transcriptomic over-expression driven by amplicons.

Consequently, we adopted a stricter and more conservative cutoff corresponding to the top 12% of the distribution (RSEM ≥ 26,641). This stringent transcriptomic threshold was subsequently applied to the GSE21050 study cohort to precisely isolate the transcriptomically over-expressed subset and evaluate its prognostic significance independent of cellular proliferation (Table A1).

For CINSARC analysis, the optimal probe set was selected using the maximum variance method, whereby the probe showing the greatest variance across all samples was selected [18]. The mean normalized expression value of the 67 selected probes was calculated for each case in GSE21050, and the population was classified into CINSARC-high and CINSARC-low groups using the median as the cutoff.

### 2.3. In Silico Screening of Candidate Genes in the 12q13-15 Amplicon

To identify candidate genes in the 12q13-15 region associated with *MDM2* expression independent of the proliferative status, we analyzed representative genes (*fibroblast growth factor receptor substrate 2* [*FRS2*], *cyclin-dependent kinase 4* [*CDK4*], *high mobility group AT-hook 2* [*HMGA2*], and *YEATS domain containing 4* [*YEATS4*]) located in the 12q13-15 region, where *MDM2* resides, as candidate genes of interest [19]. According to the human genome assembly (GRCh38), *HMGA2* (12q14.3) and *CDK4* (12q14.1) reside in the proximal region, whereas *MDM2* (12q15), *YEATS4* (12q15), and *FRS2* (12q15) form a tight gene cluster located more distally. Expression data from the GSE21050 dataset were organized and representative probe expression levels for each gene were extracted using the GEOparse library in Python (Python Software Foundation, Python 3.10, Wilmington, NC, USA). Transcript abundance was quantified using specific probe sets from the Affymetrix HG-U133 Plus 2.0 array: 203153_at (*FRS2*), 201991_s_at (*YEATS4*), 205569_at (*HMGA2*), 202246_s_at (*CDK4*), and 204439_at (*MDM2*). Subsequently, bivariate correlation analysis using Pearson’s product-moment correlation analysis was performed to evaluate the relationship between the expression level of each candidate gene and *MDM2* expression, as well as the relationship between each candidate gene and the CINSARC score. Genes showing a strong positive correlation with *MDM2* expression but no significant correlation with CINSARC were considered candidate co-associated genes, independent of proliferative status.

### 2.4. Statistical Analysis

Statistical analyses were performed to evaluate the clinical and molecular characteristics of each subgroup. Metastasis-free survival (MFS) was estimated using the Kaplan–Meier method, and differences between groups were assessed using the log-rank test. Pearson’s correlation coefficients (r) were calculated to assess the association between gene expression variables. All analyses were performed in Python using the SciPy and Lifeline statistical libraries. All *p*-values were two-sided, and *p* < 0.05 was considered statistically significant.

## 3. Results

### 3.1. Patient Characteristics and Molecular Stratification

To establish an expression-based threshold for MDM2-high tumors, RNA sequencing data from TCGA-SARC cohort were analyzed (Table A1). Tumors within the top 12% of the *MDM2* expression distribution were classified as the MDM2-high group.

**Figure 1:**
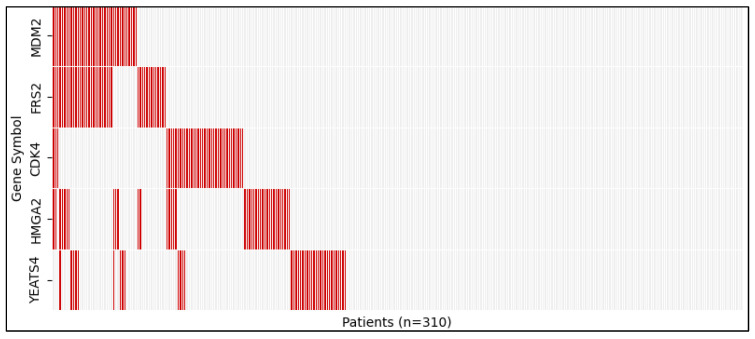
Expression patterns of representative genes in the 12q13-15 region of the GSE21050 dataset. Each column represents an individual tumor sample, and each row represents the gene. This figure illustrates the distribution of gene expression patterns across the cohort and provides a transcriptomic context for the association observed between *MDM2* and *FRS2* in the present study.

A summary plot was used to visualize the distribution of the relative expression patterns of representative genes located within the 12q13-15 region across the analyzed sarcoma cohort (Figure 1).

The 310 cases in the GSE21050 dataset were stratified into four subgroups based on MDM2 expression status and CINSARC classification. The subtype distribution was as follows: MDM2-low/CINSARC-low (*n* = 143), MDM2-low/CINSARC-high (*n* = 129), MDM2-high/CINSARC-low (*n* = 12), and MDM2-high/CINSARC-high (*n* = 26). The histological subtype distribution across these subgroups is presented in Table 1.

### 3.2. Analyses of Oncologic Outcomes with MDM2/CINSARC Stratification

**Figure 2:**
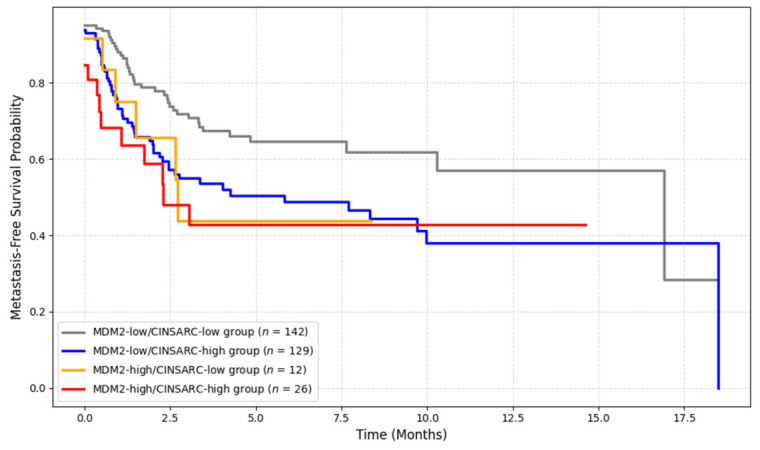
Metastasis-Free Survival by CINSARC/MDM2 Stratification.

To evaluate whether CINSARC is effective in predicting the prognosis of MDM2 expression-defined subgroups, MFS was analyzed using the Kaplan–Meier method. Among the 310 initially stratified patients, one patient was excluded from the survival analysis because of missing clinical follow-up data, resulting in the evaluation of 309 cases. The MDM2-low/CINSARC-low group showed the most favorable MFS (log-rank test, *p* = 0.00461) compared with the MDM2-low/CINSARC-high group. In contrast, the survival curve of the MDM2-high/CINSARC-low group overlapped with those of the MDM2-low/CINSARC-high and MDM2-high/CINSARC-high groups, showing no statistically significant differences among the three subgroups (log-rank test, *p* = 0.77; Figure 2). These findings suggest a trend toward unfavorable clinical behavior in a subset of MDM2-high tumors despite a low CINSARC score.

### 3.3. Transcriptomic Screening for CINSARC-Independent Candidate Genes

To investigate the molecular features underlying the poor MFS observed in both MDM2-high groups, including the MDM2-high/CINSARC-low and MDM2-high/CINSARC-high groups, we performed a correlation-based transcriptomic screening of representative genes (*FRS2*, *YEATS4*, *HMGA2*, and *CDK4*) in the 12q13-15 region in relation to *MDM2* expression and the CINSARC score (Table 2).

Across the GSE21050 dataset, *FRS2* expression strongly and positively correlated with *MDM2* expression (r = 0.760). In contrast, *FRS2* expression did not correlate with CINSARC expression (r = −0.036). *CDK4* and *HMGA2* levels did not significantly correlate with *MDM2* (r = −0.043 and r = 0.003, respectively). *MDM2*, *CDK4* and *YEATS4* were weakly correlated with CINSARC (r = 0.112, r = −0.135 and r = 0.191, respectively).

**Figure 3:**
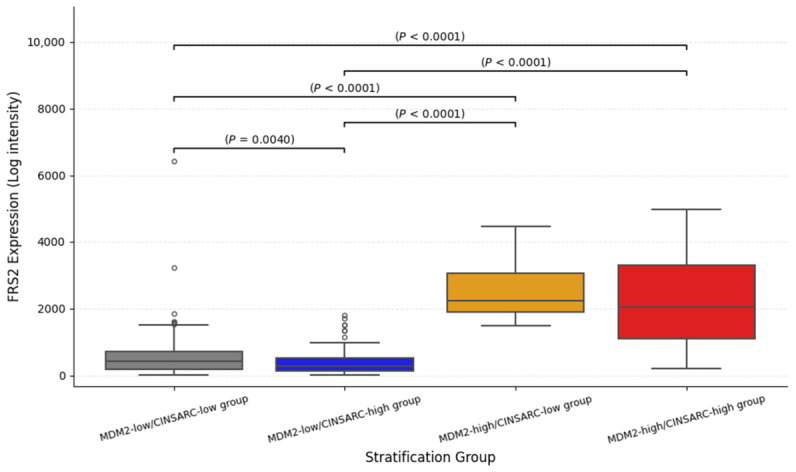
*FRS2* Expression Levels by CINSARC/MDM2 Stratification.

The distribution of *FRS2* expression in each subgroup was visualized using box-and-whisker plots (Figure 3). Marked overexpression of *FRS2* was observed exclusively in the MDM2-high group of patients. In contrast, the MDM2-low/CINSARC-high and MDM2-low/CINSARC-low groups maintained *FRS2* expression within the baseline range, suggesting that elevated *FRS2* expression was not directly associated with proliferative activity, as reflected by the CINSARC.

## 4. Discussion

In this study, we identified a subset of MDM2-high sarcomas with low CINSARC scores, in which the proliferative status and clinical outcomes appeared to be dissociated. In conventional sarcoma grading systems, tumors with low mitotic counts are classified as low-grade and are generally considered to have a favorable prognosis and indolent course [20]. Consistent with this concept, a low CINSARC score was generally associated with a favorable prognosis in the present cohort. However, the conventional assumption that low proliferative potential, as reflected by a low CINSARC score, indicated that low metastatic risk did not appear to fully apply to the MDM2-high subgroup. Our MFS data showed that the MDM2-high group, despite having a low CINSARC score, had a poor MFS risk comparable to that of the MDM2-high/CINSARC-high group. These findings highlight the possibility that a subset of MDM2-high sarcomas may have an underestimated metastatic risk when the assessment is based primarily on conventional pathological grading or proliferation-related markers such as Ki-67 [21]. Collectively, these results highlight the importance of incorporating molecular and transcriptomic stratification into future clinical studies on MDM2-high sarcomas.

Several cancer-related genes, including *CDK4*, are located in the 12q13-15 region [22]. However, the correlation analyses performed in this study (Table 2) revealed that across a wide range of soft tissue sarcomas, *FRS2* was most strongly correlated with *MDM2* expression at the mRNA level and showed no significant correlation with CINSARC, suggesting an association that is independent of the proliferative status. *FRS2* is a critical intracellular adaptor protein that serves as a hub in fibroblast growth factor receptor (FGFR) signaling pathways and regulates numerous cellular processes, including proliferation, differentiation, survival, and migration [23]. Previous studies have reported the co-amplification of *FRS2* and *MDM2* in atypical lipomatous tumors and dedifferentiated liposarcomas [23]. However, to our knowledge, this study is the first to use large-scale transcriptomic data, together with the CINSARC score, to highlight the association between *FRS2* and *MDM2* expression, which appears to be independent of the proliferative status. Given its location within the 12q13-15 region, the association between *FRS2* and *MDM2* expression may indicate a biologically relevant role beyond simply reflecting proliferation-related activity [24].

Experimental studies have shown that *FRS2* mediates FGFR signaling cascades by linking activated FGFR kinase activity to downstream pathways, including MAPK, PI3K/AKT, and PLCγ signaling, all of which are implicated in tumorigenesis and metastasis [25]. In soft tissue sarcomas, the amplification of *FRS2* and subsequent activation of the FGFR/FRS2 signaling pathway are functionally associated with the development of highly aggressive and metastatic phenotypes, particularly in high-grade liposarcomas [25]. In breast cancer models, FGFR inhibition reduces FRS2 phosphorylation and downstream signaling, resulting in decreased tumor cell proliferation, induced apoptosis, and suppressed metastatic potential [26]. Collectively, these observations suggest that FRS2-associated signaling may play a role in oncogenic processes, such as migration and metastasis, in addition to its relationship with proliferative activity.

Although FRS2 lacks intrinsic kinase activity and is therefore a challenging direct therapeutic target, its essential role as a scaffold in FGFR signaling suggests that the pharmacological inhibition of FGFR may effectively block FRS2-mediated oncogenic signaling [27]. Preclinical studies have demonstrated that the inhibition of upstream FGFR signaling using tyrosine kinase inhibitors reduces FRS2 phosphorylation and suppresses cancer cell survival and tumor progression [27]. Furthermore, FGFR-targeted therapies may overcome drug resistance driven by FGFR mutations or amplifications, which are frequently observed in multiple cancer types [28]. One possible explanation for the poor prognosis observed in the MDM2-high group may be the phenotypic transformation toward invasion and metastasis associated with FRS2-related signaling. This may explain why conventional anticancer therapies that primarily target cell proliferation are insufficient for this subset. Preclinical studies have supported this rationale, demonstrating that highly aggressive liposarcomas harboring FRS2 amplification are fundamentally dependent on the FGFR/FRS2 signaling axis and consequently exhibit significant sensitivity to FGFR inhibitors, resulting in the profound suppression of tumor growth both in vitro and in vivo [22]. In tumors with high FRS2 expression, the increased dependence on FGFR signaling may enhance the therapeutic potential of combined pathway inhibition. Specifically, simultaneous restoration of the p53 pathway through MDM2 inhibition and suppression of survival signaling through FGFR inhibition may represent a promising therapeutic strategy, regardless of the proliferation rate [22]. Patients for whom conventional cytotoxic drugs are ineffective owing to slow cell division can benefit the most from molecularly targeted therapies.

This study has several limitations. First, its retrospective design may limit the generalizability of its findings to other populations. Second, the number of cases in the MDM2-high/CINSARC-low subgroup was small, which reduced the statistical power of the subgroup-specific survival analyses and warranted cautious interpretation. Third, MDM2 status was defined using an expression-based surrogate rather than a direct assessment of gene amplification by copy number analysis or fluorescence in situ hybridization. Fourth, the analysis was based on a single public dataset without independent external validation of the results. Fifth, the histological composition of the cohort was entirely dependent on the publicly available GSE21050 dataset. Certain MDM2-amplified sarcoma subtypes, such as intimal sarcoma, were not represented, which may limit the generalizability of these findings. In fact, 27 cases were labeled as “Other” in the original GEO metadata, preventing further histopathological subclassification of this group. Finally, because the publicly available GEO annotations did not include age, sex, treatment, grade, or other detailed clinicopathological variables, a multivariate analysis was not performed to adjust for potential confounding clinicopathological factors. Furthermore, *FRS2* was evaluated only at the transcriptomic level, and no protein-level or functional validation was conducted. Therefore, prospective validations using independent clinical cohorts and experimental models are required to confirm these findings. Nevertheless, the observations in this relatively large cohort of 310 patients support the potential biological relevance of *FRS2* and justify further investigations in larger, independently validated studies. The present in silico approach should be regarded as hypothesis-generating and as providing a framework for prioritizing molecular associations, including the proliferation-independent MDM2/FRS2 axis, for subsequent pathological and experimental validation rather than replacing conventional diagnostic assessment.

## 5. Conclusions

This study suggests that proliferation-based risk assessment alone may underestimate the metastatic risk of a subset of MDM2-high sarcomas and that *FRS2* may be a biologically relevant marker associated with aggressive behavior independent of tumor proliferation. These findings confirm the potential value of integrating molecularly informed therapeutic approaches with conventional histology-based strategies in selected sarcoma subsets.

## Figures and Tables

**Table 1:** Histological Subtype Distribution across Stratified Subgroups.

Tumor Histology	Total (*N* = 310)	MDM2-Low/ CINSARC-Low Group	MDM2-Low/ CINSARC-High Group	MDM2-High/ CINSARC-Low Group	MDM2-High/ CINSARC-High Group
Undifferentiated sarcoma	136 (43.9%)	51 (35.7%)	70 (54.3%)	4 (33.3%)	11 (42.3%)
Leiomyosarcoma	85 (27.4%)	33 (23.1%)	37 (28.7%)	4 (33.3%)	11 (42.3%)
Dedifferentiated liposarcoma	62 (20.0%)	44 (30.8%)	13 (10.1%)	3 (25.0%)	2 (7.7%)
Other	27 (8.7%)	15 (10.5%)	9 (7.0%)	1 (8.3%)	2 (7.7%)
Total	310 (100%)	143 (100%)	129 (100%)	12 (100%)	26 (100%)

**Table 2:** Transcriptomic Correlation Screening of Representative Genes in the 12q13-15 Region in the GSE21050 dataset.

Genes	Correlation with *MDM2* Expression	*p*-Value	Correlation with CINSARC	*p*-Value
*FRS2*	0.760	1.11 × 10^−59^	−0.036	0.5280
*YEATS4*	0.099	0.081	0.191	0.0007
*HMGA2*	0.003	0.960	0.042	0.4620
*CDK4*	−0.043	0.454	−0.135	0.0172
*MDM2*	Reference	Reference	0.112	0.0492

## Data Availability

These data were derived from the following resources available in the public domain: https://www.ncbi.nlm.nih.gov/geo/query/acc.cgi?acc=GSE21050. accessed on 1 June 2026.

## Abbreviations

Abbreviations used in this manuscript are as follows:

*CDK4*: cyclin-dependent kinase 4; CINSARC: Complexity Index in SARComas; FGFR: fibroblast growth factor receptor; *FRS2*: fibroblast growth factor receptor substrate 2; GEO: Gene Expression Omnibus; *HMGA2*: high mobility group AT-hook 2; *MDM2*: Murine Double Minute 2 MFS: metastasis-free survival PFS: progression-free survival; RSEM: RNA-sequencing by Expectation-Maximization; TCGA: the Cancer Genome Atlas; *YEATS4*: YEATS Domain Containing 4.

## Appendix A

**Table A1:** Gaussian Mixture Model (GMM) Clustering of MDM2 Expression in TCGA-SARC Cohort.

Groups	Proportion	Mean Expression (RSEM)	Cutoff Threshold (RSEM)
Non-MDM2 amplified population (Gaussian Mixture Model)	71.7%	1083	–
MDM2-Amplified population (Gaussian Mixture Model)	28.3%	26,000	2350
MDM2-high population (this Study cut-off value)	12.0%	43,675	26,641
